# Femoral Bone Marrow Insulin Sensitivity Is Increased by Resistance Training in Elderly Female Offspring of Overweight and Obese Mothers

**DOI:** 10.1371/journal.pone.0163723

**Published:** 2016-09-26

**Authors:** Ville Huovinen, Marco Bucci, Heta Lipponen, Riku Kiviranta, Samuel Sandboge, Juho Raiko, Suvi Koskinen, Kalle Koskensalo, Johan G. Eriksson, Riitta Parkkola, Patricia Iozzo, Pirjo Nuutila

**Affiliations:** 1 Turku PET Centre, University of Turku, Turku, Finland; 2 Department of Radiology, University of Turku and Turku University Hospital, Turku, Finland; 3 Department of Endocrinology, Turku University Hospital, Turku, Finland; 4 Departments of Medicine and Medical Biochemistry and Genetics, University of Turku, Turku, Finland; 5 Folkhälsan Research Centre, Helsinki, Finland; 6 National Institute for Health and Welfare, Department of Chronic Disease Prevention, Helsinki, Finland; 7 Department of General Practice and Primary Health Care, University of Helsinki, and Helsinki University Hospital Finland, Helsinki, Finland; 8 Institute of Clinical Physiology, National Research Council (CNR), Pisa, Italy; Victoria University, AUSTRALIA

## Abstract

Bone marrow insulin sensitivity may be an important factor for bone health in addition to bone mineral density especially in insulin resistant conditions. First we aimed to study if prenatal maternal obesity plays a role in determining bone marrow insulin sensitivity in elderly female offspring. Secondly we studied if a four-month individualized resistance training intervention increases bone marrow insulin sensitivity in elderly female offspring and whether this possible positive outcome is regulated by the offspring’s mother’s obesity status. 37 frail elderly females (mean age 71.9 ± 3.1 years) of which 20 were offspring of lean/normal-weight mothers (OLM, maternal BMI ≤ 26.3 kg/m^2^) and 17 were offspring of obese/overweight mothers (OOM, maternal BMI ≥ 28.1 kg/m^2^) were studied before and after a four-month individualized resistance training intervention. Nine age- and sex-matched non-frail controls (maternal BMI ≤ 26.3 kg/m^2^) were studied at baseline. Femoral bone marrow (FBM) and vertebral bone marrow (VBM) insulin sensitivity were measured using [^18^F]fluoro-2-deoxy-D-glucose positron emission tomography with computer tomography under hyperinsulinemic euglycemic clamp. We found that bone marrow insulin sensitivity was not related to maternal obesity status but FBM insulin sensitivity correlated with whole body insulin sensitivity (R = 0.487, p = 0.001). A four-month resistance training intervention increased FBM insulin sensitivity by 47% (p = 0.006) only in OOM, while VBM insulin sensitivity remained unchanged regardless of the maternal obesity status. In conclusion, FBM and VBM glucose metabolism reacts differently to a four-month resistance training intervention in elderly women according to their maternal obesity status.

**Trial Registration:**

ClinicalTrials.gov NCT01931540

## Introduction

Maternal obesity during pregnancy increases fetal insulin resistance [1] and exposes the offspring to type 2 diabetes (T2D) especially in females [2,3]. The mother’s genetic background also interacts with intrauterine programming but this may be a less important factor that determines health in later life than maternal obesity [4]. This study is part of the EU funded DORIAN program (Developmental ORIgins of healthy and unhealthy AgeiNg) that aims to investigate the long-term impact of maternal obesity on the health of the offspring [5]. In our recent study we showed that resistance training intervention increases thigh muscle insulin-stimulated glucose uptake (GU) and whole body insulin sensitivity in frail offspring of obese/overweight mothers (OOM) [6]. However, the role that maternal obesity plays during pregnancy and the effect of resistance training on bone marrow insulin sensitivity, which may be a potential factor for bone overall health in addition to BMD, remain unknown.

Bone marrow consists of hematopoietic marrow, fatty marrow, actual bone cells and connective tissue [7]. However, very little is known about the metabolic activity of bone marrow. Muscle strength and resistance training correlate with BMD [8–11], which is associated with bone marrow fat (BMF) content [12–14]. Bone marrow is the only place in the human body in which trabecular bone and fat are directly connected [15], and this suggests that BMF may be a key regulator of trabecular bone health [16]. BMF seems to be important because it may also have an impact on whole body insulin resistance through complex cellular mechanisms [17]. Moreover it is known that high body fat content increases body overall insulin resistance [18] but it is not known whether BMF content is associated with impaired bone marrow glucose metabolism in human subjects. However, there is preliminary evidence that increased BMF content decreases vertebral bone marrow (VBM) insulin sensitivity in pigs [19].

The hyperinsulinemic euglycemic clamp is the golden standard technique to study overall insulin sensitivity [20]. [^18^F]FDG-PET (fluoro-2-deoxy-D-glucose positron emission tomography), in combination with mutual hyperinsulinemic euglycemic clamp, can be used to study tissue-specific insulin sensitivity, for example, in bone marrow. BMF content can be measured with magnetic resonance proton spectroscopy (^1^H-MRS) [21].

In this study we extend upon our previous findings related to thigh muscle and whole body insulin sensitivity [6]. First, we aimed to investigate the role that prenatal maternal obesity plays in bone marrow insulin sensitivity in elderly female offspring. Secondly, we aimed to investigate if resistance training affects femoral bone marrow (FBM) and VBM insulin sensitivity in these offspring and whether this outcome is regulated by their mother’s obesity status. We hypothesized that prenatal maternal obesity is related to decreased bone marrow insulin sensitivity in elderly female offspring. We also hypothesized that resistance training increases FBM and VBM insulin sensitivity and this is greater in OOM compared to offspring of lean/normal-weight mothers (OLM) due to maternal obesity exposed insulin resistance [1]. To address these questions, we used PET-imaging with a glucose analogue, fluoro-2-deoxy-D-glucose (FDG), tracer under insulin-stimulated circumstances to assess bone marrow glucose metabolism in frail offspring with a history of their maternal obesity status. Imaging studies were carried out before and after a four-month individualized resistance training intervention.

## Material and Methods

### Study subjects

Subjects were recruited at the Folkhälsan Research Center, Helsinki, Finland between 21^st^ of May 2012 and 4^th^ of December 2013 from the Helsinki Birth Cohort Study (HBCS) II [2], which included 13345 subjects born during 1934 to 1944. The eligible study subjects were selected from a sub-cohort of 2003 subjects that have been intensively clinically characterized throughout the years. Detailed inclusion and exclusion criteria have been described earlier [6]. Based on the last study visit, all potential subjects were listed and contacted by study nurse (P. Nyholm). Altogether 46 eligible women subjects aged 68–78 years agreed to participate and were successfully recruited in the study. 20 women were selected as frail and offspring of lean/normal-weight mother (OLM) participants, 17 women as frail and offspring of obese/overweight mother (OOM) participants and nine women as non-frail and OLM control participants. Frail subjects belonged to the lower half of handgrip strength values and control subject belonged to the upper half of handgrip strength values, as measured from all HBCS participants in 2001–04. The handgrip test has been used as a valid marker for detecting frail subjects in elderly populations [22]. Data for the handgrip strength measurement was obtained from the earlier conducted clinical study [23]. Offspring of lean/normal-weight mothers (OLM) had a prenatal maternal BMI ≤ 26.3 kg/m^2^ (lowest two quartiles of the entire population) and offspring of obese/overweight mother (OOM) had a prenatal maternal BMI ≥ 28.1 kg/m^2^ (highest quartile of the entire population). BMI of the participant’s mothers were measured prior to delivery. The BMI was calculated as weight (kg) divided by height squared (m^2^).

Subjects with diabetes requiring insulin treatment or fasting glucose more than 7 mmol/L at the last visit before the enrollment were excluded. Subjects currently smoking or with comorbidities influencing insulin sensitivity and with contraindications for participating in an exercise intervention (i.e., chronic atrial fibrillation and pacemaker) or an MRI study were also excluded. No differences were found in age (p = 0.22) or BMI (p = 0.56) between included and excluded women subjects. Sample size was based on our previous study investigating the effect of resistance training on thigh muscle and whole body insulin sensitivity [6]. We decided that a clinically significant effect of 25% or greater would be of interest. To achieve this we calculated that with two-tailed alpha level of 0.05 and with power of 0.8 a total of approximately 40 subjects (20 OLM and 20 OOM) would be needed if tested with paired samples t-test, which was used to test effects within the groups in our previous study [6].

### Study design

**Fig 1:**
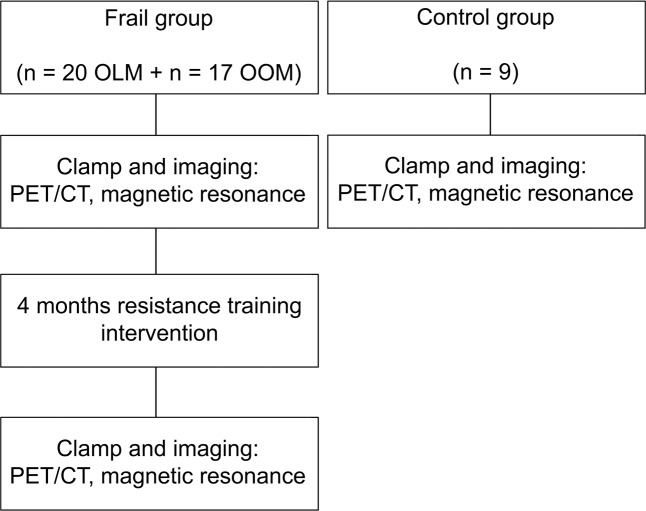
Study design. OLM (offspring of lean/normal-weight mothers), OOM (offspring of obese/overweight mothers). Modified from [6].

The study design is depicted in Fig 1 [6]. The frail subjects were studied before and after the intervention. The control subjects were studied only at baseline. Studies took place at the Turku PET Centre, University of Turku, Turku, Finland between 8^th^ of June 2012 and 29^th^ of April 2014. After the first study visit, the frail subjects underwent an individualized four-month resistance training intervention three times a week, 60 minutes per session under supervision of a trained, licensed physiotherapist for four months in Folkhälsan Research Center’s gym. Resistance training sessions started with warm-up (10 minutes) with a cycle and/or elliptical ergometer. It continued with eight different resistance exercises targeting large muscle groups of the lower and upper body (e.g., leg presses, chest presses, seated rows, abdominal crunches, back extensions, seated leg extensions, seated leg curls and hip abductions). At each station, subjects completed three sets of 8–15 repetitions with a load that corresponded to 50–80% of estimated 1 repetition maximum (RM). One RM for each exercise was estimated from 8-RM tests by applying Epley’s formula [24]. Progress in muscle strength was measured once a month and the loads for the following month were adjusted as appropriate.

Adherence (actualized individual exercise frequency per week divided by maximum exercise amount per week) to exercise was 76.3 ± 11.1% in OLM and 80.8 ± 10.3% in OOM (p = 0.23). There was no difference in total number of exercise sessions (p = 0.37), leisure-time physical activity frequency (p = 0.92) or duration (p = 0.82) between OLM and OOM during the intervention. Two frail subjects did not participate in the intervention. Thus, the number of subjects was lower in the frail group after the intervention (n = 35). In addition, one subject withdrew from postintervention PET-imaging and technical difficulties were encountered during postintervention PET-imaging regarding two subjects. ^1^H-MRI spectroscopy of the vertebral bone marrow was done for the subset of 28 subjects before the intervention and for the subset of 29 subjects after the intervention. Four frail and two control subjects used metformin regularly. However, the medication for diabetes was temporarily discontinued 48 hours before PET-scanning. The ethics committee of the Hospital District of Southwestern Finland approved the studies (26/180/2012), which were conducted according to the principles of the Declaration of Helsinki. All subjects gave their written informed consent before imaging studies. The study is registered to Clinicaltrials.gov (NCT01931540).

Studies were performed after an overnight (12 hours) fasting and they included PET-CT imaging session, an MRI imaging session and clinical examination performed by physician (VH, HL, JR, SK) which included sitting blood pressure from the left arm [Omron M3 HEM-7200-E2], measurements of waist and hip circumference, weight, height, total body fat percentage [Omron HBF-400-E], fasting serum insulin [automatized electro-chemiluminescence immunoassay, ECLIA; Cobas 8000, Roche Diagnostics GmbH, Mannheim, Germany] and fasting plasma glucose [glucose oxidase method, Analox GM9 Analyzer, Analox Instruments Ltd. London, U.K]. Maternal obesity status of the subjects was not known by the investigators during the studies.

### MRI-imaging

Magnetic resonance spectroscopy was performed on the bone marrow of the 10th thoracic vertebral body. Typical acquisition parameters for the PRESS sequence were: TR/TE 5000/25, bandwidth 1kHz, number of data points 2048, number of acquisitions 32, voxel size 15 x 18 x 15 mm. A single element of Sense Body coil was used for the signal reception.

LCModel (version 6.3-0C, protocol ‘lipid-4’ for lipid quantification) [25] was used for the analysis of the spectra. Triglyceride signals from around 0.9 to around 2.0 ppm were considered as the "fat" signal since water signal overlapped the resonances around 5.3 ppm. Both fat and water resonance areas were corrected for the T2 relaxation effect using the T2 times (75.4 ms for fat and 26.9 ms for water) measured by Kugel et al [26]. T1 effects were considered to be negligible because of the long duration of the repetition. T2 corrections were performed using the equation SI = SI_0exp(-TE/T2) where SI is the corrected and SI_0 the uncorrected resonance area.

Fat-fraction [27] was calculated by dividing the T2 corrected resonance area of fat by the sum of T2 corrected areas of water and fat.

### PET-imaging

All participants underwent abdominal and thigh PET-imaging with fluoro-2-deoxy-glucose ([^18^F]FDG) in an overnight (12 hours) fasting state during hyperinsulinemic euglycemic clamp with a GE Advance PET scanner (Discovery 690, General Electric (GE) Medical systems, Milwaukee, WI, USA). ^18^F-FDG was synthesized with a computer-controlled apparatus in accordance with a modified method described by Hamacher et al [28]. After 90 minutes of hyperinsulinemia, an [^18^F]FDG bolus (188 ± 10 MBq) was injected through the cannula on the left antecubital vein. Forty minutes after the injection, the upper abdomen and lumbar spine were imaged for 15 minutes and 55 minutes after the injection thigh area and diaphysis of the femoral bone were imaged for 15 minutes (frames, 5 x 180 seconds). Hyperinsulinemic euglycemic clamp (1 mU/kg * min, Actrapid; Novo Nordisk) was performed as previously described [29] and the whole-body glucose uptake (M-value) representing systemic insulin sensitivity was calculated from the glucose infusion rates during 60–120 minutes of the clamp.

### PET/CT-image processing and analysis

**Fig 2:**
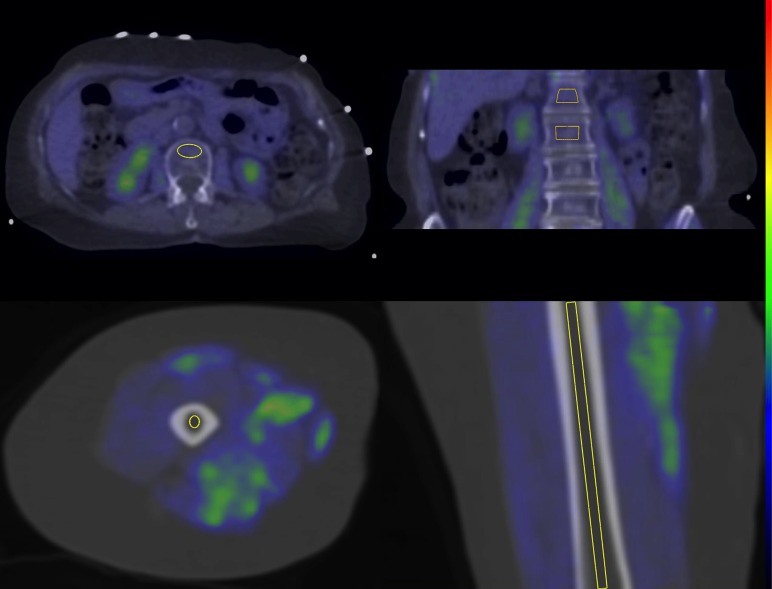
Cylinder-shaped volumes of interests of vertebral (upper row) and femoral bone marrow (lower row) in transaxial and coronal PET/CT-images.

All data was corrected for time decay, dead time and measured photon attenuation. Data was reconstructed with GE iterative VuePoint method and with an axial slice thickness of 3.75 mm. Volumes of interest (VOIs) were drawn on the diaphysis of the right femoral bone marrow and on the lumbar bone marrow (L1-L2) excluding the cortex (Fig 2), on the psoas major muscle and on subcutaneous adipose tissue of the hip using Carimas 2.7 software (Turku PET Centre). CT-images were used as an anatomical reference. Time activity curves were obtained as described in our previous article [6]. The transfer rate of [^18^F]FDG from plasma into tissue was calculated from the time-activity curves in plasma and using a Gjedde-Patlak plot as a graphical analysis [30]. Tissue glucose uptake was obtained after multiplying the transfer rate with mean plasma glucose concentration divided by lumped constant, which was set to 1.0 for bone marrow, 1.2 for skeletal muscle [31] and 1.14 for subcutaneous adipose tissue [18]. Maternal obesity status of the subjects was not known by the investigators during the PET/CT-image processing and analysis.

### Statistical analyses

**Fig 3:**
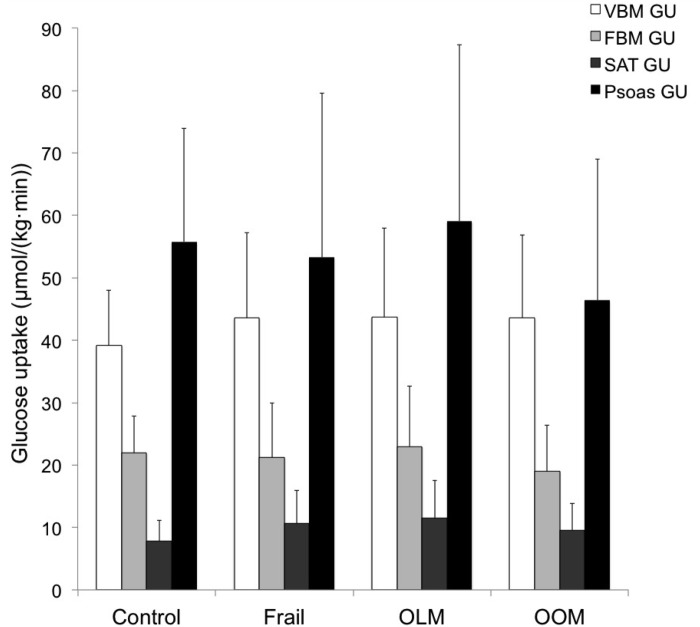
Tissue-specific insulin-stimulated glucose uptake of control, frail (OLM + OOM), OLM and OOM at baseline. At baseline, there were no differences between the groups. VBM: vertebral bone marrow, FBM: femoral bone marrow, Psoas: psoas muscle, SAT: subcutaneous adipose tissue, GU: glucose uptake, OLM: offspring of lean/normal-weight mothers, OOM: offspring of obese/overweight mothers. Results are shown as mean ± SD.

**Fig 4:**
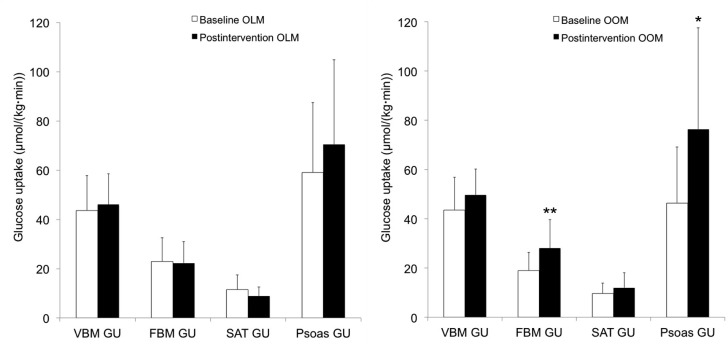
Insulin-stimulated glucose uptake (GU) at baseline and after the intervention in (left) offspring of lean/normal-weight mothers (OLM) and (right) in offspring of obese/overweight mothers (OOM). There was no change in any tissue-specific GU after intervention in OLM. FBM GU (p = 0.006**) and psoas muscle GU (p = 0.039*) increased significantly after the intervention in OOM. Results are shown as mean ± SD.

Statistical analyses were performed with SPSS software for MacOS (version 22.00). Data is presented as mean ± standard deviation (SD). The Shapiro–Wilk test was used to assess the normality of variables. Differences between the groups at baseline (results of Table 1 and Fig 3) were tested with parametric ANOVA (age, BMI, body fat %, systolic BP, diastolic BP, fP-glucose, maternal BMI, handgrip strength, VBM fat/water -ratio, M-value, VBM GU, FBM GU, psoas GU) or with Kruskal-Wallis test (waist/hip ratio, fS-insulin, SAT GU). ANCOVA was used to adjust for the possible effect of muscle strength at baseline. The effects of exercise between and within the groups were assessed with repeated measures ANOVA as a linear mixed model with unstructured covariance structure (results of Table 2 and Fig 4). Bonferroni adjusted p-values were used in pairwise comparisons between the time points. Differences between categorical data were tested with Chi-square test. Associations of bone marrow insulin sensitivity, the main outcome variable, with other variables were tested with Spearman (ΔFBM GU vs ΔM-value in OOM, ΔVBM vs ΔM-value in OOM) or Pearson (all the other correlation testing) correlation analysis. A p-value of less than 0.05 was considered significant.

**Table 1:** Participants anthropometric and biochemical characteristics at the baseline.

	Control (n = 9)	Frail (n = 37)	OLM (n = 20)	OOM (n = 17)
**Age (years)**	71.4 ± 3.1	71.9 ± 3.1	72.3 ± 2.6	71.5 ± 3.7
**BMI (kg/m**^**2**^**)**	27.8 ± 4.2	27.2 ± 4.7	26.6 ± 4.8	27.9 ± 4.6
**Body fat %**	40.1 ± 4.9	39.5 ± 5.9	38.6 ± 6.6	40.5 ± 4.9
**Systolic BP (mmHg)**	156 ± 13	162 ± 16	161 ± 12	163 ± 19
**Diastolic BP (mmHg)**	87 ± 11	90 ± 10	88 ± 10	93 ± 9
**Waist/hip ratio**	0.90 ± 0.03	0.91 ± 0.05	0.91 ± 0.05	0.90 ± 0.04
**fP-glucose (mmol/L)**	6.4 ± 0.4	6.0 ± 0.7	6.0 ± 0.7	5.9 ± 0.8
**fS-insulin (mU/L)**	8.8 ± 3.6	8.5 ± 4.3	9.6 ± 3.7	9.4 ± 4.9
**Handgrip strength (kg)**^**1**^	29.7 ± 2.6	17.7 ± 2.6*	17.2 ± 2.7*	18.2 ± 2.5*
**Maternal BMI (kg/m**^**2**^**)**	23.8 ± 2.0	26.0 ± 3.7	22.9 ± 1.4	29.7 ± 1.6*†
**VBM fat/water -ratio (%)**	44.4 ± 6.6 ^a^	43.8 ± 7.3 ^b^	46.0 ± 7.0 ^c^	42.2 ± 7.3 ^d^
**M-value (μmol/(kg·min))**	22.3 ± 10.3	23.9 ± 10.6	26.5 ± 11.8	20.9 ± 8.3
**T2D (subjects/%)**	2 (22.2%)	4 (10.8%)	3 (15.0%)	1 (5.9%)

Results are presented as mean ± SD. Frail: OLM (Offspring of lean/normal-weight mothers) + OOM (Offspring of obese/overweight mothers). Comparisons between control vs frail, control vs OLM, control vs OOM and OLM vs OOM are assessed. VBM: vertebral bone marrow, M-value: whole body insulin sensitivity, T2D: type 2 diabetes. Modified from [6].

^1^ Assessed in 2001–2004

* p < 0.001 vs control

† p < 0.001 vs OLM

^a^ n = 7

^b^ n = 21

^c^ n = 9

^d^ n = 12

**Table 2:** Baseline and postintervention characteristics of offspring of lean/normal-weight mothers (OLM) and offspring of obese/overweight mothers (OOM).

	OLM			OOM		
	Baseline (n = 20)	Exercise (n = 19)	p	Baseline (n = 17)	Exercise (n = 16)	p
**BMI (kg/m**^**2**^**)**	26.6 ± 4.8	27.1 ± 4.7	0.55	27.9 ± 4.6	27.6 ± 4.8	0.96
**Body fat %**	38.6 ± 6.6	38.8 ± 6.3	0.48	40.5 ± 4.9	39.8 ± 4.5	0.19
**Systolic BP (mmHg)**	161 ± 12	154 ± 15	0.07	163 ± 19	158 ± 16	0.28
**Diastolic BP (mmHg)**	88 ± 10	84 ± 11	0.14	93 ± 9	89 ± 9	0.21
**Waist/hip ratio**	0.91 ± 0.05	0.89 ± 0.05	0.10	0.90 ± 0.04	0.88 ± 0.06	0.27
**fP-glucose (mmol/L)**	6.0 ± 0.7	6.1 ± 0.7	0.44	5.9 ± 0.8	5.8 ± 0.6	0.66
**fS-insulin (mU/L)**	9.6 ± 3.7	9.5 ± 4.1	0.79	9.4 ± 4.9	9.7 ± 5.6	0.32
**VBM fat/water-ratio (%)**	46.0 ± 7.0 ^a^	44.4 ± 6.1 ^b^	0.25	43.7 ± 7.2 ^c^	43.0 ± 6.2 ^d^	0.85

Results are presented as mean ± SD. VBM: vertebral bone marrow. Modified from [6].

^a^ n = 9

^b^ n = 17

^c^ n = 12

^d^ n = 12

## Results

### Baseline characteristics

Handgrip strength value assessed in 2001–2004 was significantly lower (p < 0.001) in all frails, the OLM and the OOM compared to controls. Maternal BMI was significantly higher (p < 0.001) in OOM compared to OLM and control. There were no differences in age, BMI, body fat %, systolic and diastolic blood pressure, waist-hip ratio, plasma fasting glucose, serum fasting insulin, vertebral bone marrow (VBM) fat/water ratio, whole body insulin sensitivity (M-value) or prevalence of T2D between control vs frail, control vs OLM, control vs OOM and OLM vs OOM (Table 1).

### Glucose uptake was higher in vertebral bone marrow (VBM) compared to femoral bone marrow (FBM)

VBM GU was 100% higher than FBM GU (42.8 ± 12.9 vs 21.4 ± 8.2, p < 0.001) and interestingly, the level of FBM GU was similar to body overall insulin sensitivity (M-value) (21.4 ± 8.2 vs 23.6 ± 10.4) in frails and controls combined. Even after adjusting for muscle strength, there were no differences in VBM GU, FBM GU, subcutaneous adipose tissue (SAT) GU or psoas GU between control vs frail, control vs OLM, control vs OOM or OLM vs OOM (Fig 3).

### FBM GU was associated with overall body insulin sensitivity (M-value) in OLM but not in OOM

With frails and controls combined, M-value associated strongly with FBM GU (R = 0.487, p = 0.001) but not with VBM GU. VBM fat/water-ratio and VBM GU associated inversely (R = -0.411, p = 0.030) in frails and controls combined. When the data was analysed based on the maternal obesity content, FBM GU correlated with M-value only in OLM (R = 0.693, p = 0.001) but not in OOM. As expected from the pooled analyses, a correlation between VBM GU and M-value was not observed in OLM or OOM.

### Effect of resistance training on anthropometric and biochemical characteristics

No significant changes in anthropometric or biochemical characteristics were observed between or within the groups (Table 2). VBM fat/water-ratio remained unchanged.

### Resistance training increased FBM but not VBM insulin sensitivity in OOM

In OOM, there was a significant increase (47%) in FBM GU (p = 0.006) after the resistance training intervention. Similarly, psoas GU (p = 0.039) increased while VBM GU and SAT GU remained unchanged after the intervention. In OLM, there were no significant changes in tissue-specific GU (Fig 4) or M-value. Differences in exercise effects were found in FBM GU (p = 0.022) and SAT GU (p = 0.021) between OLM and OOM.

### Change in FBM GU (ΔFBM GU) was associated with a change in body overall insulin sensitivity (ΔM-value) in both OOM and OLM

In OOM, ΔFBM GU correlated with ΔM-value (R = 0.601, p = 0.039) but not with change in anthropometric characteristics or glycemic state indicators. Similar to OOM, in OLM ΔFBM GU correlated with ΔM-value (R = 0.717, p = 0.001). There was no correlation between ΔVBM GU and ΔM-value in OOM or OLM.

## Discussion

We studied the effect of maternal obesity and resistance training on femoral bone marrow (FBM) and vertebral bone marrow (VBM) insulin sensitivity in elderly women with the history of their mother’s obesity status. Our main finding was that a four-month individualized resistance training intervention is sufficient to significantly improve insulin sensitivity in FBM in the offspring of obese/overweight mothers (OOM). Resistance training also improved psoas muscle insulin sensitivity in OOM, while it had no effect on any GU values in offspring of lean/normal-weight mothers (OLM) or on VBM insulin sensitivity in OOM or OLM. This is in line with our previous work in which, in the same study population, resistance training improved thigh muscle insulin-stimulated GU and whole body insulin sensitivity (M-value) in OOM but not in OLM [6]. However, we cannot completely exclude the possibility that increase in psoas GU in this study or thigh muscle GU in our previous study would have been significant also in OLM with a larger sample size. In a pooled analysis, M-value significantly correlated with FBM insulin sensitivity but not with VBM insulin sensitivity. In addition, the training-induced improvement in FBM insulin sensitivity positively correlated with the change in M-value in both OOM and OLM. Our findings, together with previous knowledge that in the elderly, FBM contains mostly fat [32], demonstrates that FBM is an insulin sensitive organ, its insulin sensitivity very closely follows whole body insulin sensitivity and if insulin resistance has developed, it can be alleviated by resistance training in a relatively short time in OOM. These findings highlight the novel role that FBM adipose tissue plays as a metabolically active fat depot.

The most interesting finding in our study was that resistance training improved FBM insulin sensitivity in OOM but not in OLM. Resistance training has also earlier been observed to be beneficial for tissue-specific GU. Reichkendler et al. investigated the effect of an 11-week aerobic training intervention on site-specific insulin sensitivity in young male subjects. They found that muscle GU increased [33] and this was also the case considering psoas muscle GU in OOM in our study. The difference between the effects of exercise on FBM and VBM GU may originate from the simple fact that FBM contains more fat than VBM (40–50% in our study). Nonetheless, the underlying mechanism behind the FBM insulin sensitivity improvement is unknown. In adults, FBM contains mostly fat that may become insulin-resistant [32,34]. The increase in FBM GU correlated with an increase in M-value. It may be that maternal obesity blunts FBM GU initially but the effect of resistance training proves beneficial for FBM GU in OOM. One explanation could be that maternal obesity alters adipocyte or whole body metabolism leading to a different behavior of FBM GU between OLM and OOM. Samuelsson et al. suggested in their mice study that a mechanistic role for the maternal obesity-induced offspring insulin resistance could be developmentally programmed physical inactivity or altered adipocyte metabolism [35]. Another explanation could be that femoral bone marrow may contain more fat in OOM than in OLM. However, femoral BMF content was not measured in our study and to our knowledge no studies investigating the effect of maternal obesity on femoral BMF content have been conducted.

In our study, VBM fat/water-ratio was between 40–50% in every group at baseline and differences were not observed between the groups indicating that maternal obesity is not related with VBM fat content. Moreover, resistance training did not have a different effect on the VBM fat content than the intramyocellular lipid content in the thigh muscle that was reduced after resistance training in the OOM group, in our previous work [6]. This means that VBM fat content and intramyocellular lipid content seem to react differently to resistance training. Interestingly, we found an inverse association between VBM fat/water-ratio and VBM GU. This is in accordance with our previous pilot study with obese diabetic pigs in which VBM GU inversely associated with VBM fat content, while there was no difference in BMF content or VBM GU between these pigs and healthy control pigs [19]. These data suggest a distinct regulation of glucose metabolism at different bone marrow sites and FBM seems to be insulin sensitive while VBM appears to be insulin resistant. According to our results, VBM GU was 1.5–2 times higher than FBM GU, which could be at least partly explained by the relatively high proportion of metabolically very active hematopoietic red marrow in VBM [36]. This data warrants further studies into the glucose metabolism of hematopoietic bone marrow and for discovering the possible link between bone marrow glucose metabolism and BMD especially in insulin resistant conditions such as type 2 diabetes (T2D).

A major strength in our study was the [^18^F]FDG-PET imaging modality that allowed us to study site-specific insulin sensitivity. In addition, the hyperinsulinemic euglycemic clamp is a valid mean to investigate total body insulin sensitivity [20] and it was performed during [^18^F]FDG -PET-study to standardize the insulin action. Our study population consisted of elderly female subjects from a very extensive longitudinal cohort. This gave us the unique chance to study the role that maternal obesity plays in bone marrow insulin sensitivity. Implication of our work is particularly important because, it may offer new insights into more individualised and more accurately allocated lifestyle interventions in treating or preventing frailty or T2D in patients with known maternal prenatal obesity status.

We also recognize some weaknesses in our study. According to our results, there were no significant differences in whole body, psoas muscle or bone marrow insulin sensitivity between OLM and OOM at baseline unlike we expected. However, thigh muscle insulin sensitivity was significantly lower in OOM than in OLM at baseline as shown in our recent report from the same cohort [6]. Skeletal muscle GU is the main contributor of whole body insulin sensitivity and therefore an optimal tissue to assess differences between the groups [29]. A weakness for the studies on bone marrow metabolism was that the sample size was relatively low due to limitations in study design and imaging techniques used. Therefore, it is not excluded that differences would have been observed in FBM and psoas muscle GU between OLM and OOM at baseline if the sample size was larger. Moreover, not to find differences between OLM and OOM may be due to the fact that the mothers were only moderately obese, which may originate from the lower prevalence of obesity during 1934 to 1944. During pregnancy women gain weight very differently, which means that pre-deliver BMI may possibly be misleading as a health outcome predictor. However, our outcomes refer to maternal gravidic BMI, since pre-pregnancy BMI is not available in the HBCS cohort. Our results in a more recent unpublished cohort (n = 83) by Eriksson JG et al show that pre-pregnancy and end-pregnancy BMI correlate strongly (R = 0.94, p < 0.001). In addition, we have recently shown [2] that gravidic BMI is a significant predictor of chronic diseases in the elderly offspring in the HBCS. However, we cannot rule out that the use of pre-pregnancy BMI as stratification criterion may have led to a different outcome in the current study.

Frailty is known to be associated with insulin resistance [37] but we did not find any differences in whole body, psoas muscle or bone marrow insulin sensitivity between frails and controls. This may depend on the fact that our definition of frailty was not multifactorial. We based our definition of frailty only on handgrip strength, which has been considered a valid marker for frailty [22,38]. Thus our subjects could be considered as “semi-frail.” Nonetheless, our main aim was to study the effect of both maternal obesity and a resistance training intervention on bone marrow GU. Even though our definition of frailty would have been multifactorial or there would have been a difference in whole body insulin sensitivity between control and frail group, the sub-grouping for maternal obesity would still have shown differences between OLM and OOM after the intervention. The weaknesses mentioned above probably influence the external validity of our results. OLM and OOM were similar considering T2D status in contrast to the fact that maternal obesity exposes to T2D [2]. It may be that bigger sample sizes are needed to show differences in T2D prevalence. The control group was studied only at baseline and hence we cannot know how the resistance training intervention would have affected the tissue-specific and overall GU in controls.

In conclusion, this was the first study to investigate the role that maternal obesity plays in bone marrow insulin sensitivity in elderly women offspring. We also assessed the effect of resistance training on this insulin sensitivity. We found that four-month individualized resistance training increased FBM insulin sensitivity in OOM but not in OLM, which may reflect altered maternal obesity induced bone marrow adipocyte metabolism. However, VBM insulin sensitivity was not affected regardless of offspring maternal obesity status during pregnancy. Our finding demonstrates that FBM is an insulin sensitive fat depot and that the glucose metabolisms of FBM and VBM are regulated differently. This may reflect the distinct ability to consume glucose between fatty and hematopoietic bone marrow, which may be a crucial factor for overall bone health in addition to BMD.

## Supporting Information

S1 File CONSORT 2010 Flow Diagram. (DOC)

S2 File TREND Statement Checklist. (PDF)

S3 File Clinical Study Protocol. (DOC)
