# Supplementary material for: Femoral Bone Marrow Insulin Sensitivity Is Increased by Resistance Training in Elderly Female Offspring of Overweight and Obese Mothers
Source: PLoS One. 2016 Sep 26;11(9):e0163723. doi: 10.1371/journal.pone.0163723 (PMC5036877; doi:10.1371/journal.pone.0163723)
Supplement: S1 File — (DOC) [file pone.0163723.s001.doc]

**
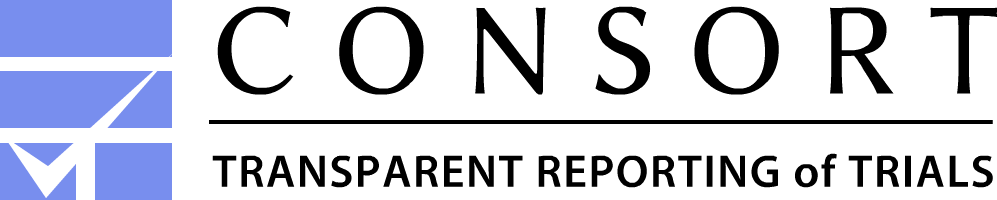
**

**CONSORT 2010 Flow Diagram**

**Allocation**

**Analysis**

**Follow-Up**

**Enrollment**

Assessed for eligibility (n=46)

Excluded (n= 0)

♦  Not meeting inclusion criteria (n=0)

♦  Declined to participate (n=0)

♦  Other reasons (n=0)

Analysed (n=37)
♦ Excluded from analysis (n=0)

Lost to follow-up (give reasons) (n=0)

Discontinued intervention (Did not participate) (n=2)

Allocated to intervention (n=37)

♦ Received allocated intervention (n=37)

♦ Did not receive allocated intervention (give reasons) (n=0)

♦ Did not receive allocated intervention (In our study setting controls did not receive intervention) (n=9)

Analysed (n=9)
♦ Excluded from analysis (n=0)

Randomized (n=0)
