# Supplementary material for: Femoral Bone Marrow Insulin Sensitivity Is Increased by Resistance Training in Elderly Female Offspring of Overweight and Obese Mothers
Source: PLoS One. 2016 Sep 26;11(9):e0163723. doi: 10.1371/journal.pone.0163723 (PMC5036877; doi:10.1371/journal.pone.0163723)
Supplement: S3 File — (DOC) [file pone.0163723.s003.doc]

Turku PET Centre

Turku University Central Hospital

**CLINICAL STUDY PROTOCOL**

**Version:** 1.4 (07.03.2013)

**Study ID:** **DORIAN**

**Study title:** Developmental ORIgins of healthy and unhealthy AgeiNg:

the role of maternal obesity

**Phase:** not applicable

**PET:**  [^18^F]FDG (18F-fluorodeoxyglucose)

**Principal investigators:**

**PET part : Intervention part :**

Pirjo Nuutila Johan Eriksson

Turku PET Centre Samfundet Folkhälsan i svenska Finland rf (Folkhälsan)

and University of Helsinki

at Turku University Central Hospital Dept of General Practice

Kiinamyllynkatu 4-8 and Primary Health Care

P.O. Box 52, FIN-20520 Turku, Finland P.O. Box 20, FIN-00014 Helsinki, Finland

Phone : +358-2 2611 868 Phone: +358-9-19127557

Fax : +358-2-2318191 Fax : +358-9-19127536

Email : pirjo.nuutila@utu.fi Email : johan.eriksson@helsinki.fi

Department of General Practice and Primary Health Care, University of Helsinki, P.O Box 20, Helsinki 00014, Finland

PET group: Pirjo Nuutila, Marco Bucci, Ville Huovinen, Virva Lepomäki, Anna Karmi, Riitta Parkkola, Lauri Nummenmaa, Riku Kiviranta, Tam Pham, Henry Karlsson, Patricia Iozzo

Intervention group: Johan Eriksson, Samuel Sandboge, Taina Rantanen, Katri Räikkönen, Liisa Penttinen, Mika Simonen, Minna Salonen, Paula Nyholm, Terttu Nopanen

SIGNATURES

Principal investigator: __________________________ Date: ___/___/___

Name

Responsible hospital

physicist: __________________________ Date: ___/___/___

Name

Director of Turku

PET Centre: __________________________ Date: ___/___/___

Name

**TABLE OF CONTENTS**

1 sUMMARY [4](#__RefHeading___Toc317774436)

2 INTRODUCTION [4](#__RefHeading___Toc317774437)

3 OBJECTIVES AND HYPOTHESES [7](#__RefHeading___Toc317774438)

4 STUDY DESIGN [8](#__RefHeading___Toc317774439)

**4.1 Study type** [8](#__RefHeading___Toc317774440)

**4.2 Study design** [8](#__RefHeading___Toc317774441)

5 PATIENT/SUBJECT SELECTION [8](#__RefHeading___Toc317774442)

5.1 Source population [8](#__RefHeading___Toc317774443)

5.2 Number of subjects [9](#__RefHeading___Toc317774444)

5.3 Inclusion criteria for all subjects (frail and controls) [9](#__RefHeading___Toc317774445)

5.4 Inclusion criteria for the frail population [9](#__RefHeading___Toc317774446)

5.5 Inclusion criteria for the control population [9](#__RefHeading___Toc317774447)

5.6 Exclusion criteria for the the whole study population (55 subjects) [9](#__RefHeading___Toc317774448)

6 ASSESSMENTS [10](#__RefHeading___Toc317774449)

6.1 General study outline [10](#__RefHeading___Toc317774450)

6.1.1 Description of the intervention [10](#__RefHeading___Toc317774451)

6.3 PET studies [11](#__RefHeading___Toc317774452)

6.3.1 Description of the method [11](#__RefHeading___Toc317774453)

6.4 CT studies [13](#__RefHeading___Toc317774454)

6.5 US studies [13](#__RefHeading___Toc317774455)

6.6 MRI/MRS/fMRI studies [13](#__RefHeading___Toc317774456)

6.7.1 1H MRS and MRI studies [13](#__RefHeading___Toc317774457)

6.7.2 Functional MRI (fMRI) studies [15](#__RefHeading___Toc317774458)

6.8 Euglycemic hyperinsulinemic clamp [17](#__RefHeading___Toc317774459)

6.9 Bioimpedance [17](#__RefHeading___Toc317774460)

6.10 Inflammatory and epigenetic biomarkers from subcutaneous adipose tissue biopsy [18](#__RefHeading___Toc317774461)

6.11 LaboratORy measurEmEnts, **aDdendum 03/2013** [18](#__RefHeading___Toc317774461)

7 SAFETY [18](#__RefHeading___Toc317774462)

7.1 Safety measurements [18](#__RefHeading___Toc317774463)

8 THE CURRENT PHASE OF THE STUDY [19](#__RefHeading___Toc317774464)

9 REFERENCES [19](#__RefHeading___Toc317774465)

# 1 sUMMARY

The prevalence of obesity in the developed world has increased markedly over the last 20 years. Considering the prevalence of obese and overweight adult subjects, and the fact that pregnancy itself induces a state of insulin resistance and inflammation, maternal obesity may be the most common health risk for the developing fetus [1]. It is well established that what we eat has a major impact on our health. However, there is growing evidence to suggest that diet during pregnancy and lactation may be particularly important as not only does it influence the health of the mother, it may have a permanent effect on the health of her children and even her grandchildren. The concept that environmental factors, such as nutrition during early development, influence both our health span and lifespan has been termed the developmental origins of health and disease hypothesis [2].

The objective of the study is to examine if signs of frailty (condition developed with ageing) can be reversed by lifestyle induced modifications of its primary components (IR, sarcopenia, psychological profile) in offspring of overweight/obese vs lean mothers. Subjects will undergo characterization of biohumoral markers, a 75 g oral glucose tolerance test, imaging biomarkers (PET/CT, US, fMRI-MRS), genetic biomarkers (DNA and telomere damage) and inflammatory biomarkers (macrophage infiltration) before and after the 4-month lifestyle intervention period (physical exercise). By PET/CT it will be measured tissue-specific IR in skeletal muscle, adipose tissue, liver, intestine, myocardium and targeted brain regions. MRS will be used to measure organ steatosis in the skeletal muscle and liver, MRI will be used to measure fat masses in the visceral and subcutaneous abdominal areas, and skeletal muscle masses in the legs, and fMRI will be performed to assess activation in brain regions regulating cognition and appetite/energy control. US will be used to assess cardiovascular markers (IMT, strain and function). Absolute and body weight normalized values as well as fat/muscle mass ratios will indicate the diagnosis of sarcopenic obesity. Frailty related osteopenia will be measured via CT. CT will be used to measure fat masses around the heart and calcium score.

The study consists of 40 frail old subjects, age ≥ 65 sub-grouped in 20 offspring of overweight and obese mothers during pregnancy, and 20 offspring of lean mothers and 15 non frail controls. These subjects will be studied with positron emission tomography (PET), computed tomography (CT) and magnetic resonance imaging (MRI) and spectroscopy (MRS). In addition functional MRI (fMRI) will be performed. Subjects will be physically examined and anthropometric data will be measured along with blood sampling.

# 2 INTRODUCTION

Europe currently has the highest proportion of elderly people in the world and is expected to maintain this leading position for the next 50 years. Though a series of markers and genes implicated in longevity have been identified, the increase in longevity has not been accompanied by an increase in disease-free life expectancy, i.e. compressed morbidity. CVD, T2D, sarcopenia and cognitive decline are highly prevalent in the elderly. These disorders share common early risk factors, they are mutually reinforcing [3], and frequently coexist in the same aged individual, and with mood disorders. This clustering of different conditions deteriorates quality of life in the elderly. Chronic diseases start at a young age and only manifest themselves clinically at more advanced age.

The age-related vulnerability towards these chronic diseases originates from a common pathogenic platform of cumulative damage [3-5]. Ageing takes place in different ways between individuals, with varying rates for different organ systems, and it can range from healthy to pathological. Therefore, a life course approach to the study of ageing is crucial. In this continuum, the term “frailty” is used to indicate the intermediate condition, and imply a certain reversibility of pathological processes. Recognized features of frailty include muscle wasting/fatigue, insulin resistance and central fat accumulation, inflammation and oxidative stress (OS), mood changes and a reduced adaptation to stressful or novel situations [5].

Knowledge of biological mechanisms occurring during the early stages of life, including pre- and perinatal phases, are important for understanding/predicting changes affecting health during the life course. The concept of fetal programming, i.e. that an insult during organ development leads to permanent disease susceptibility during adult life, shares remarkable similarities with ageing, because both situations are characterized by a limited capacity to compensate and repair, due to system exhaustion in ageing and system immaturity in fetal programming. Healthy pregnancy is a state of hepatic and adipose tissue IR, low-grade inflammation, visceral fat accumulation, dyslipidemia and prothrombosis. The limited data so far available in humans indicate that the fetus of obese mothers [6;7] is exposed to higher levels of insulin, due to maternal IR, increasing neonatal fat mass, whereas the ensuing foetal IR [8] compromises the development of skeletal muscle, heart, brain, pancreas. The placenta in obese mothers expresses higher levels of proinflammatory mediators, and dysfunctional adipokine and vascular patterns [9]. Overall, gestational IR and obesity favour hyperglycaemia, relative fat excess and sarcopenia, greater birth weight and higher blood pressure in the offspring, which are recognized risk factors for CVD, T2D, and cognitive impairment. We hypothesize that maternal obesity leads to fuel mediated teratogenesis, reflecting a particularly surreptitious form of premature ageing, characterized by features of frailty.

IR is defined as the decreased biological response of a nutrient to a given concentration of insulin at the target tissue, e.g. liver, muscle, adipose tissue, brain, heart, endothelium and it is tethered to hyperinsulinaemia. The impairment of insulin-mediated blood flow and glucose uptake in skeletal muscle and adipose tissue, and suppression of hepatic glucose production and adipose tissue lipolysis contributes to the pathogenesis of hyperglycaemia, and exacerbates IR [10]. The enlargement of adipocytes, leading to adipose tissue hypoxia and dysfunction, immune cell infiltration, apoptosis, and inflammatory adipocytokine secretion is considered a potential initiator of the metabolic disturbances of IR [11;12]. Compensatory hyperinsulinaemia stimulates lipogenesis, ectopic lipid accumulation, and enhanced hepatic triglyceride release, proliferation of vessel smooth muscle cells and atherosclerosis, and renal sodium retention and hypertension. Hyperglycaemia and dyslipidemia may contribute to the endothelial dysfunction which is typically associated with IR states [13]. Myocardial IR is characterized by reduced cardiac perfusion and impaired glucose uptake [14;15], both of which have negative prognostic implications, compromising the adaptability of the heart to work stress and oxygen depletion. In the brain, IR translates in alterations of glucose metabolism and an impaired suppression of appetite. We have shown that an abnormal brain glucose exposure occurs in IR, non-diabetic obese rats [16] and humans with the metabolic syndrome [17]. IR is also negatively related with the volume of the hippocampus, a brain region in which glucose disposal is connected with memory skills [18].

Physiological states associated with energy balance are primary determinants of eating behaviour. Comparative studies have identified an interconnected network comprised by amygdala, ventral striatal and midbrain regions in various aspects of food reward processing [19-21]. Measuring brain responses with functional MRI (fMRI) while viewing food images can thus be used to study how the combined lifestyle intervention modifies the responsiveness of the reward system, which in turn will provide the foundations for a neural model of energy balance control in the brain.

Lifestyle interventions delay the development of age-related diseases [22] and are important in improving the quality of life.

Positron emission tomography (PET) combined with [^18^F]-2-fluoro-2-deoxy-D-glucose ([^18^F]FDG) and the model of Sokoloff [23] have been validated and used to measure regional glucose uptake in the heart and skeletal muscle [24]. [^18^F]FDG is transported to heart cell and skeletal muscle cell and phosphorylated. In contrast to glucose, it cannot be further metabolised and it remains trapped in the cytosol [23;25]. With [^18^F]FDG it is possible to study glucose transport and phosphorylation. With euglycemic hyperinsulinemic clamp it is possible to study the target organ in a steady state condition of insulin stimulation and evaluate organ insulin resistance [26].

# 3 OBJECTIVES AND HYPOTHESES

The main hypotheses of the PET studies are that

The glucose uptake (FDG) among the 40 frail elderly subjects

- 1. in insulin-sensitive organs is decreased in offspring of overweight/obese mothers compared to lean;
  2. in offspring of overweight/obese mothers the intervention will be more effective in increasing the organ glucose uptake.

The glucose uptake (FDG) of 40 frail elderly subjects compared to control subjects

- 1. will be reduced in the insulin stimulated state because of a probable insulin resistance condition.

The main hypotheses of MRI/MRS

We predict that the fat organ content, especially liver fat will be decreased after intervention, especially in the subgroup of offspring of overweight/obese mothers.

The main hypotheses of CT

We predict that the frail subjects will present a osteopenic/sarcopenic state as compared to controls and that intervention might improve the muscle/bone ratio. We expect to find enhanced accumulation of fat surrounding the heart in offspring of overweight/obese mothers.

The main hypotheses of the brain activation studies (fMRI):

We predict to find enhanced reward responses to external food cues in offspring of overweight/obese mothers.

The main hypotheses of US

We predict that cardiac function will be compromised in the frail group and that it will improve after the intervention.

The objectives of this study are to measure the possible differences between frail offspring of overweight/obese and lean mothers and differences between frail subjects and controls; finally, to evaluate the effect of a secondary prevention (lifestyle intervention) programme:

1. On whole body and regional insulin stimulated glucose metabolism in myocardium, skeletal muscle, liver, pancreas, targeted brain regions, subcutaneous and visceral fat, intestine and bone.
2. On total body fat and abdominal fat distribution & abdominal fat volumes.
3. On the fat/muscle mass and bone/muscle mass ratios.
4. On skeletal muscle, liver, heart and pancreas fat content.
5. On glycemic control and on biochemical markers (HDL, LDL, triglycerides, cholesterol).
6. On inflammatory and epigenetic markers present in subcutaneous adipose tissue.
7. On brain white and gray matter volumes and distribution.
8. On brain regions regulating cognition, stress responses and appetite/energy control.
9. On brain activation response to food stimuli before and after the intervention.
10. On correlation between the subjective feelings of hunger and brain activation.

# 4 STUDY DESIGN

## **4.1 Study type**

The study is a cohort prospective study. The centres involved in this study are Turku PET Centre, University of Turku, Samfundet Folkhälsan i svenska Finland rf (Folkhälsan) (Helsinki) and University of Helsinki. The combined lifestyle intervention is described in section 6.1.1.

## **4.2 Study design**

The study includes two time points (at baseline and after intervention) where 40 elderly subjects will be studied using PET/CT/MRI/MRS/fMRI/US. The PET examination requires an overnight fasting (8-12 hours). First some anthropometric measurements are taken, and then the PET exam will start. The subjects are canulated and the euglycemic hyperinsulinemic clamp starts at -40 min from the injection of the ^18^F-FDG. The PET/CT exam lasts approximately 100 min (+40 to achieve the steady state with the clamp). After the PET/CT study, subjects are allowed to rest an have a light meal. After this the echocardiographic exam is performed and followed by the MRI-MRS and finally fMRI examinations.

# 5 PATIENT/SUBJECT SELECTION

## 5.1 Source population

##

The study subjects will be recruited from the Helsinki Birth Cohort Study II (HBCS II), the largest, best-characterized longitudinal cohort in the world. HBSC II includes 13345 subjects born in 1934-1944. The study subjects will be selected from a sub-cohort of 2003 subjects that have been deeply clinically characterized throughout the years.

## 5.2 Number of subjects

##

The study in Turku consists of 40 elderly **frail** subjects, 20 offspring of overweight/obese mothers and 20 offspring of lean mothers and 15 elderly non-frail control subjects. **Frailty** has many definitions, one of the first widely accepted was proposed by Fried et al. including criteria such as unintentional weight loss, loss of muscle mass (sarcopenia), weakness (low grip strength), poor endurance and exhaustion (self-reported), slowness (short walking time). One of the mainly recognized components is the muscle weakness derived; for this reason these one parameter will be the main selection criteria. The frail subjects will be study twice, at baseline and after about four months of treatment, while controls will be studied only at baseline.

## 5.3 Inclusion criteria for all subjects (frail and controls)

Age: 68-78 years

Gender: Female

## 5.4 Inclusion criteria for the frail population

1. Lower half of adult grip strength (measured 2001-2004)

40 subjects subgrouped in

1. 20 offspring of overweight/obese mothers (highest quartile)
2. 20 offspring of lean mothers (lowest two quartiles)

## 5.5 Inclusion criteria for the control population

Non-frail and offspring of normal weight mothers

## 5.6 Exclusion criteria for the the whole study population (55 subjects)

1. Oral corticosteroidal or Varfarin therapy
2. Recent myocardial infarction
3. Severe chronic disorder that can prevent to participate the intervention
4. Chronic atrial fibrillation and pacemaker
5. Cancer less than 5 years ago
6. Current smoking
7. Diabetes requiring insulin treatment or fasting glucose more than 7 mmol/l
8. Weight more than 170 kg and Waist circumference > 150 cm
9. Inner ear implants
10. Metal objects in body including metallic prostheses, artificial valve prostheses, surgical clipses, braces, foreign fragments or tattoo

# 6 ASSESSMENTS

## 6.1 General study outline

All measurements will be performed before and after a four-month lifestyle intervention programme carried out in Helsinki. A brief description of the intervention follows.

## 6.1.1 Description of the intervention

The subjects will participate in a 4-month individualized progressive circuit-type resistance training programme three times a week, under the supervision of a physiotherapist. The sessions will include aerobic warm-up on a bicycle. The programme consists of 8 different movements per circuit; two sets of 10-12 repetitions will be performed at each station. Each station exercise a different large muscle group (e.g. thigh flexors and extensors, trunk flexors and extensors, upper arm muscles), alternating between upper and lower body. The subjects will move from station to station in a continuous fashion with short rests (<60 s) between the stations. Based on the one repetition maximum (1 RM) or a modest intensity (with the previously mentioned number of repetitions), according to the Borg scale, the start-up intensity of the training will be set. As strength will improve, the intensity of the programme will be progressively increased. No other changes in exercise habit will be made on behalf of the study group.

The programme is according to general exercise recommendations for the general population including elderly subjects.

6.2 Measurements

All the measurements included in the study performed in Turku are listed below:

1. Anthropometric measurements;
2. Glycaemic control, hormones and biochemical markers (partly in Helsinki);
3. Body fat distribution, Fat/Skeletal muscle ratio;
4. Skeletal muscle/Bone ratio and Bone density;
5. Fat volumes (subcutaneous and visceral adipose tissue);
6. Organ fat content (skeletal muscle, liver);
7. Inflammatory and epigenetic markers from a subcutaneous adipose tissue biopsy;
8. Whole-body and regional insulin stimulated glucose metabolism in myocardium, skeletal muscle, liver, pancreas, brain, intestine, bone and subcutaneous and visceral fat;
9. Cognition, stress responses and food-stimuli mediated brain activation (a subgroup filling the criteria of fMRI);
10. Brain white & grey matter volumes (a subgroup filling the criteria of MRI);
11. Cardiovascular markers (IMT, strain and function).

## 6.3 PET studies

PET studies will be performed twice: at baseline and after four-month treatment. PET studies consist of a single PET session where glucose metabolism in the myocardium, skeletal muscle, pancreas, liver, intestine, bone, brain, subcutaneous and visceral adipose tissue is measured during euglycemic hyperinsulinemic clamp [26] using [^18^F]FDG PET method. It will be possible to evaluate differences between offspring of overweight/obese mothers and lean mothers in terms of organ insulin resistance.

## 6.3.1 Description of the method

Studies are performed after a 10-12 hour overnight fast. Two catheters are inserted, one in an antecubital vein for injection of glucose, insulin, and for tracer injections and another in the opposite antecubital arterialized vein for blood sampling. The subjects will be lying in a supine position throughout the study.

[^18^F]FDG is synthesized with an automatic apparatus (Hamacher). The [^18^F]FDG-study is performed according to the protocol. Patient is positioned into the camera. Attenuation correction is performed. ~5 mCi (180 MBq) of [^18^F]FDG is injected intravenously over 2 min. The PET-scanning starts from the

liver and heart, followed by legs, abdomen and brain. All scans are dynamic.

|  | 🡻 [^18^F]FDG 180 MBq bolus | | | | | | | |
| --- | --- | --- | --- | --- | --- | --- | --- | --- |
| Start  of the  clamp | CT-scan  heart,liver+  Calcium S.  6 min | FDG  heart  liver  40 min | CT-scans  abdomen  + lumbar  6 min | FDG  abdomen  15 min | CT-scans  legs  3 min | FDG  legs  15 min | CT-scan  brain  3 min | FDG  brain  15 min |
|  |  |  |  |  |  |  |  |  |
| -40 | -6 | 0 | 40 | 46 | 61 | 64 | 79 | 82 97 |
|  |  | Frames  **8x 15s**  **3x 60s**  **7x 300s** |  | Frames  **5x 180s** |  | Frames  **5x 180s** |  | Frames  **5x 180s** |

An integrated PET/CT, GE Discovery^TM^ ST System (General Electric Medical Systems, Milwaukee, WI, USA) with resolution of 3.75 mm is used for PET studies. All data will be corrected for dead-time, decay and measured photon attenuation. Dynamic PET-scans will be reconstructed with MRP reconstruction method [27]. Brain scan is performed with a covering the whole brain in 3D mode. CT scans will be conducted to correct for photon attenuation and to evaluate fat masses and tissue density [28]. Plasma radioactivity is measured with an automatic gamma counter (Wizard 1480 3", Wallac, Turku, Finland). Heart rate and blood pressure will be monitored during the studies to calculate the rate-pressure product. Plasma glucose and free fatty acids are frequently measured during the PET study.

PET images are analysed using standard procedures. In short, the images are analysed using VINCI software (Max-Planck Institute for Neurological Research, Cologne, Germany). Regions of interest (ROIs) are manually drawn over the analysed region. Time-activity curves (TACs) are generated from all ROIs for further analysis: dynamic images are further analysed using Patlak plot analysis and static images using an approximation of Patlak (fractional uptake rate, i.e. FUR). When needed anatomical reference images from CT are used to localise the area of interest. When possible compartmental modelling will be utilized for advanced kinetic analysis [29].

Dosimetry calculations are the following:

- PET: 180 MBq (FDG) x 0.019 mSv/MBq = 3.42 mSv (x 2 = 6.84 mSv)

- CT: attenuation scans (4 scan (3 low/1 highres)/ session) = 0.3 x 3 + 0.6 = 1.5 mSv (x 2 = 3 mSv)

- CT: calcium score = 1 mSv (x 2 = 2 mSv)

- CT: for bone density measurement (1 scan/session) = 2 mSv (x 2 = 4 mSv)

The total dose accounting all PET/CT-studies performed before and after the treatment is 15.84 mSv. It must be considered that adults of 65 years old have 5 to 10 times lower risk to develop fatal cancer from the radiations; even if 15.84 mSv is comparable to less than 4 years of background radiation, the risk of incurring in fatal cancer to correspond to the risk of dying when driving 23 to 116 thousand kilometres or to smoking 8 to 18 packs of cigarettes [30;31].

## 6.4 CT studies

CT will be used to assess bone density and evaluate the frailty-related osteopenia via a dedicated scan at the spine (lumbar region) in presence of a calibration phantom. Calcium Score and fat masses around the heart will be measured as previously described [32].

## 6.5 US studies

##

Ultrasound studies (US) will be performed to measure cardiac function, intima-media thickness (IMT) at the carotid level, and strain.

## 6.6 MRI/MRS/fMRI studies

Changes in body fat volumes & distribution and ectopic fat are measured using MRI and MRS. Abdominal, femoral, thoracic and cerebral areas are imaged with MRI. Brain imaging is used for the measurement of white and grey matter volumes. Areas of thalamus, hypophysis and pituitary are of special interest in the brain scan. Liver, skeletal muscle, and brain are analyzed with magnetic resonance spectroscopy. Data from MRI/MRS/fMRI studies is obtained using either 1.5 Tesla – or 3.0 Tesla system (Intera, Philips Medical Systems, Best, the, Netherlands).

## 6.7.1 1H MRS and MRI studies

*Liver studies*

Axial T1-weighted dual fast field echo images (TE 2.3 and 4.6 ms, TR 120 ms, slice thickness 10 mm without gap), covering the area of the liver are acquired during standardized breath-hold instructions. A MR imager (Gyroscan Intera CV Nova Dual, Philips Medical Systems, The Netherlands) with a flexible surface coil and body coil is used for MRI and MRS. A single voxel with a volume of 27 cm^3^ is positioned in the liver outside the area of the great vessels. To ensure similar voxel placement before and after the intervention, the voxel location is recorded in each patient. A PRESS 1H MRS sequence is used with the following parameters: TR = 3000 ms, TE = 25 ms with data acquired during breath-hold intervals. 1H MRS findings of the liver have been validated in both animal and human studies [33;34]. Using a local workstation, liver margins are outlined manually on each individual image. Total liver volume is calculated by multiplying the measured surface areas of each slice by the slice thickness, as previously described [35].

*Adipose tissue*

T1W FFE images are obtained in the abdominal region to measure the adipose tissue masses (subcutaneous and visceral) via dedicated software (Slice-o-Matic, Tomovision, Canada).

*Skeletal muscle studies*

A single voxel with a volume of 1.8 cm^3^ was positioned in the tibialis anterior muscle to quantify the intramyocellular and extramyocellular lipid contents (IMCL, EMCL) and creatine-to-water ratios. To ensure identical voxel placement before and after intervention, voxel location was graphically recorded in each patient. A PRESS 1H-MRS sequence was used with the following parameters: TR = 3,000 ms, TE = 27 ms. All spectra were analyzed using LCModel (33). The lipid and water amplitudes were corrected due to different T-2 decay and molar concentrations of 1H nuclei in fat and water (35). 1H-MRS findings of IMCL content have been validated in both animal and human studies (41).

MRI of the femoral region will be used to assess the bone-to-muscle ratio.

*Brain studies*

The following conventional image sequences are acquired: (1) sagittal T1-weighted sequence (TR/TE 650/15 [repetition time/echo time msec]), (2) coronal fluid attenuated inversion recovery (FLAIR) sequence (TR/TE/TI 11000/140/2800 [repetition time/echo time/inversion time msec]), (3) transverse T2-weighted spin-echo sequence (TR/TE 4500/100 [repetition time/echo time msec]). All these sequences are acquired with slice thickness of 5 mm and 1 mm interslice gap. Moreover a transverse T1-weighted turbo gradient-echo sequence (TR/TE 25/4.6 msec, flip angle of 30º, contiguous sections and section thickness of 1mm) is obtained. Cerebral white matter signal intensity changes are rated on FLAIR images by using the scale proposed by Fazekas et al [36], which has a maximum score of 6 and has shown a “very good” inter-observer reproducibility in a prior study [37].

Brain diffusion tensor imaging (DTI)

For DTI, 32 non-colinear directions of gradients are acquired to obtain the whole diffusion tensor with an echo-planar imaging (EPI) single-shot sequence. Diffusion imaging acquisition parameters are as follows: TE/80 ms, 30 ms, 45 ms, 32 transverse slices, slice thickness 5 mm with a gap of 0.5 mm. ROIs are placed individually for each patient by one experienced neuroradiologist blinded to the diagnosis of morbid obesity. Contamination of adjacent structures and T2-visible focal lesions are avoided. ROI:s are placed in the following areas bilaterally: frontal, parietal, occipital white matter as well as middle thalamus, pulvinar and putamen. ROIs are also placed in the genu and splenium of corpus callosum. The FA and MD values of each ROI are measured by using Pride software (Philips Medical Systems, Best, The Netherlands). Brain volume index (BVI) is calculated from MD maps by dividing the brain volume by the sum of brain volume and cerebrospinal fluid (CSF) volume. Brain tissue and CSF are separated by using a threshold value of 2.4 x 10^-3^ mm2/s i.e. pixels with MD value of 2.4 x 10^-3^ mm2/s or more were considered as CSF.

Brain ^1^H MR spectroscopy

Using the obtained images, a single voxel for spectroscopy is positioned in the left thalamus or chemical shift imaging (CSI) grid covering a larger part of the brain. Voxel dimensions are 15 mm x 15 mm x 15 mm. A water suppressed PRESS 1H MRS sequence is used with the following parameters: TR = 2000 ms and TE = 144 ms. The total scan time is approximately 4 minutes 56 seconds. All spectres are analysed using LCModel.

## 6.7.2 Functional MRI (fMRI) studies

The main interest is whether the lifestyle intervention can specifically reduce the responses to appetizing, high-calorie foods. Scanning sessions will take place in the afternoon between 3-5 pm. The methodology is the same previously reported [38].

Echo-planar imaging (EPI) protocol with Philips Gyroscan Intera 1.5T scanner will be used. Functional and anatomical volumes are collected with Philips Gyroscan Intera 1.5T CV Nova Dual scanner. High-resolution, anatomical images (1 mm^3^ resolution) will be acquired using a T1-weighted sequence (TR=25 ms, TE=4.6 ms, flip angle 30º, scan time 376 s). Whole-brain functional volumes are acquired using blood oxygenation level dependent (BOLD) -weighted EPI sequence (TR=2998 ms, TE=50 ms, flip angle 90º, FOV=192 mm, matrix=64x64, bandwidth=62.5 kHz, slice thickness=4.0 mm, gap between slices=0.5 mm, 30 interleaved slices acquires in ascending order) sensitive to BOLD contrast. Manual responses will be acquired with a MRI compatible button box.

The stimuli consists of digitalized full-colour photographs depicting

1. Appetizing, high-calorie foods (e.g. chocolate cake, sweets)
2. Bland, low-calorie foods (e.g. cabbage, lentils)
3. Non-food objects (e.g. cars, tools)

Fifty images from each category will be used. Stimulus presentation will be controlled with Presentation computer program (Neurobehavioral Systems Inc.) Stimuli are projected from an LCD projector onto a non-magnetic screen mounted at the foot of the scanner tube, and an angled mirror reflects the images on the screen to the participants’ field of vision.

The experiment will run with a classic blocked (box-car) design. Appetizing foods, bland foods and non-food objects will be presented in separate 15 second -blocks that consist of presentation of five pictures from the respective category. A 15 s rest period (fixation) will be intermixed between the stimulation blocks to reduce the activation of the reward systems back to baseline level and to enhance the power of the design. Each stimulus will be displaced slightly to the left or to the right from the centre of the screen and the participant has to detect the direction of the displacement and respond with a button press. This ensures that the participant has to pay attention to the stimuli. A total of 10 blocks per condition will be run, and the order of the blocks will be counterbalanced across the participants. The experiment lasts about 15 minutes. After the experiment, the participants will rate how appetizing the food stimuli were, using a scale ranging from 1 (not appetizing at all) to 10 (extremely appetizing).

SPM5 software ([www.fil.ion.ucl.ac.uk/spm/software/spm5](http://www.fil.ion.ucl.ac.uk/spm/software/spm5)) is used for the data analysis [39]. First, functional images are sinc (sine cardinal) interpolated in time to correct for slice time differences and realigned to the first scan by rigid body transformations to correct head movements. Next the images are unwarped and a mean functional image is generated. The mean functional images are inspected for excessive signal dropout. EPI and structural images are co-registered and normalized to the T1 standard template in MNI space [40] using linear and non-linear transformations, and smoothed with a Gaussian kernel with 8 mm FWHM. A random effects model is implemented using a two-stage process of within (first level) and between (second level) –subjects modelling. This random-effects analysis assesses effects on the basis of inter-subject variance and thus allows making inferences from the population from where the participants were selected. For each participant a General Linear Model (GLM) is used to assess regional effects of task parameters on BOLD indices of activation. The model includes four experimental conditions (appetizing foods, bland foods, objects, rest) and effects of no interest (realignment parameters) to account for motion-related variance. Low-frequency signal drift is removed using a high-pass filter (cut-off 128 seconds) and AR(1) modelling of temporal autocorrelations is applied. The individual voxel-wise t-contrast images are generated using the following contrasts:

1. Appetizing vs. bland foods
2. Bland vs. appetizing foods
3. Appetizing and bland foods vs. objects

These images are subsequently entered into a second-level model, subjected to a voxel-wise contrast and t-test using Gaussian Random Field Theory to assess whether or not the reward systems respond to high and low calorie foods in the experimental and the control group. A mixed ANOVA will be used to assess whether the responses of the reward system are different between offsprings of overweight/obese vs lean mothers group. The appetizing vs. bland foods contrast is used to assess the activation of the reward system in the groups.

## 6.8 Euglycemic hyperinsulinemic clamp

Clamp technique is a method for quantifying insulin secretion and resistance [26]. In euglycemic hyperinsulinemic clamp, plasma insulin concentration is acutely raised and then gradually decreased until the final infusion speed has been achieved. The idea is to block hepatic glucose production. The plasma glucose concentration is kept constant at 5 mmol/l by a variable glucose infusion using a negative feedback principle. Under this steady-state condition of euglycemia, the glucose infusion rate equals glucose uptake by all the tissues in the body and is therefore a measure of tissue sensitivity to exogenous insulin. The more insulin sensitive the study subject is, the more glucose has to be infused in order to keep plasma glucose level constant.

Plasma glucose is measured before clamp. Clamp-infusate is prepared individually for each patient according to the body surface area (BSA). BSA can be calculated as followed: BSA = (weight (kg)^ 0.425) * (height (cm)^ 0.725) * 0.007184. Insulin infusion is started with priming dose 480 ml/h for 0-4 minutes. After 4 minutes insulin infusion is changed to 240 ml/h for 4-7 minutes. At the same time glucose infusion is started. If plasma glucose is normal glucose infusion rate is calculated as followed (ml/h): 0.5 * weight (kg). During clamp plasma glucose levels are constantly measured (every 5 minutes) and written down. Patient wellbeing is carefully monitored. Blood samples are withdrawn to measure insulin and FFA levels during the study. After PET, insulin infusion is stopped. Glucose infusion is continued until a stable level of plasma glucose is achieved (healthy individuals: 6.5 mmol/l). In addition a lunch is served.

The method of insulin-glucose clamp has been documented in detail, validated and described in Turku PET center’s internal method documents (METs).

## 6.9 Bioimpedance

##

Bioimpedance is done for the measurement of body fat content using electrical scale (Omron BF400). Bioimpedance is based on measuring electrical signals passing through the fat, lean mass and water in the body. The actual impedance or conductivity of various tissues in the body is known by measuring current between two electrodes and applying this information to complex proven scientific formulas, so that body composition (i.e. body fat content) can be determined.

##

## 6.10 Inflammatory and epigenetic biomarkers from subcutaneous adipose tissue biopsy

A subcutaneous adipose tissue sample will be taken and splitted into two samples. One sample will be destined to the telomere length assay, as result from the ratio between telomere repeats to SCG copies (T/S ratio) and early DNA damage by assessing the formation of γ-H2A.X positive nuclei.

Another sample will be used to investigate the inflammatory markers, such as the infiltration of macrophages into the histological sample.

6.11 Laboratory measurements

Plasma glucose is determined using the glucose oxidase method (Analox Glucose analyser, GM9). Insulin is measured using electroluminescence immunoassay (Roche Modular E170 analyzer; Roche Diagnostics GmbH, Mannheim, Germany). Serum FFAs are measured enzymatically (Wako Nefa C kit; Wako Chemicals GmbH, Germany).

**ADDENDUM 03/2013**

In addition to the above mentioned laboratory measurements, HbA1c, ALAT, ASAT, TG, HDL, and OGTT will measured either in Turku or in Helsinki. HbA1c is determined using HPLC, ASAT and ALAT are determined using photometric methods, TG and HDL are also measured via photometric methods, and OGTT is measured using a standard 75 g oral glucose tolerance test. These measurements - together with diagnosis of the metabolic syndrome, diabetes diagnosis, as well as measurement of fasting insulin, and fasting glucose – will be used to calculate the fatty liver score and equation[41], algorithm test for the diagnosis of non-alcoholic fatty liver disease. The algorithm tests have previously been applied on in the HBCS (manuscript under review) and the current study’s liver imaging offers the opportunity of validating the algorithms.

# 7 SAFETY

## 7.1 Safety measurements

Heart rate, blood pressure and plasma glucose are monitored during the studies. Chemical purity of [^18^F]FDG is measured before administration.

# 8 THE CURRENT PHASE OF THE STUDY

Started.

# 9 REFERENCES
